# Machine learning vs. radiomics for discriminating atypical cartilaginous tumors from benign enchondromas on MRI

**DOI:** 10.1186/s12938-026-01547-0

**Published:** 2026-03-24

**Authors:** Simon Johannes Joham, Johannes Nikolaus Woltsche, Dieter Szolar, Andreas Leithner, Martin Urschler, Maria Anna Smolle

**Affiliations:** 1 Institute for Medical Informatics, Statistics and Documentation, Medical University of Graz, Auenbruggerplatz 2/V, 8036 Graz, Austria; 2 Department of Orthopaedics and Trauma, Medical University of Graz, Graz, Austria; 3 Diagnostikum Graz, Graz, Austria

**Keywords:** Enchondroma, Atypical cartilaginous tumors, Magnetic resonance imaging, Artificial intelligence, Segmentation, Radiomics

## Abstract

**Background:**

Enchondromas (EC) present cartilaginous tumors that are difficult to differentiate from their intermediate counterpart, atypical cartilaginous tumors (ACT). Histologically, tumor distinction of these entities is limited by sampling bias, while radiologically, similar lesion features render classification challenging. Therefore, the aim of this study is to investigate whether machine learning- or radiomics-based image analysis tools can reliably differentiate between EC and ACT using MRI data and corresponding expert annotations.

**Methods:**

Based on an MRI dataset of 206 unique patients (79 controls, 104 EC, 23 ACT), we develop a machine learning-based AI image analysis tool that uses the state-of-the-art nnU-Net framework for medical image segmentation and extends it for tumor classification. Two nnU-Net models (Scout and Specialist) are applied sequentially. Scout first detects images without tumor tissue and removes them from further analysis, whereas Specialist performs the final tumor classification on the remaining images. Alternatively, our tool supports radiomics-based classification using hand-crafted tumor characteristics.

**Results:**

In our cross-validation experiments, when using the two models approach, where Specialist follows Scout, we achieved 87% Sensitivity (95% CI [0.67, 0.96]) for the ACT class and 93% Sensitivity (95% CI [0.87, 0.97]) for the EC class. Furthermore, no image containing an ACT was classified as non-tumor.

**Conclusions:**

In this pilot study, we demonstrated that MRI information alone can be used to differentiate between ACT and EC with high accuracy. These results seem promising that in future, machine learning and AI can be used for better orthopedic diagnosis of cartilaginous tumors in clinical practice.

**Supplementary Information:**

The online version contains supplementary material available at 10.1186/s12938-026-01547-0.

## Introduction

Benign cartilaginous tumors, i.e., enchondroma (EC), and their malignant counterpart chondrosarcoma (CS) constitute primary bone tumors [1]. CS are further subdivided into different gradings [2], with grade 1 CS of the appendicular skeleton recently being classified as “atypical cartilaginous tumor” (ACT) [3]. EC and ACT constitute frequent incidental findings on X-ray projections, magnetic resonance imaging (MRI), and computed tomography (CT) scans in musculoskeletal radiology. Their combined prevalence is 0.4% around the shoulder [4], 1.5% around the knee [5], 0.7% around the proximal femur [6] (mainly EC), and 0.07% around the hand [7] (mainly EC). Both entities share common features on imaging, including their metaphyseal location, popcorn-like calcification pattern, typical appearance on MRI (T1 hypointense, T2 hyperintense), and no soft tissue component [8].

Histopathologically, EC are lobulated tumors of low cellularity with a hyalinized matrix, with embedded chondrocytes lacking cytological atypia [8, 9]. On the other hand, ACTs are rather cell-rich and show entrapment of bony trabeculae [8, 9]. However, histology itself can be prone to sampling bias given that an area non-representative of the least differentiated tumor component may be selected for biopsy. The radiological differentiation between EC and ACT is likewise difficult, though especially when lesions are present in the long tubular bones [10]. Some imaging features have been postulated to distinguish both entities, including size (5 cm as threshold), presence and extent of endosteal scalloping, growth over time, and perilesional edema [11–13].

While EC are considered benign, ACTs may progress, and eventually dedifferentiate into higher-grade chondrosarcomas. Therefore, close monitoring or surgical removal (i.e., curettage) is recommended in the latter [14]. Other than EC and ACT, chondrosarcomas constitute malignant mesenchymal tumors with a potential to metastasize, requiring aggressive treatment with wide tumor resection, and in selected cases (e.g., non-resectable lesions) systemic chemotherapy and radiotherapy (although they are largely considered chemo- and radio-resistant). The difficulty lies in identification of atypical features indicative of semi-malignant ACTs based on imaging, in order to select the right patients for close surveillance or surgery. In this respect, artificial intelligence (AI)-based image investigation may be of value. Rather than being biased on prior knowledge and clinical reasoning, automated image analysis based on machine learning, more specifically deep learning [15] and radiomics [16, 17], has the potential to identify tumors suspicious of ACT by deciphering individual feature patterns within a lesion that eventually remain concealed from the human eye. Moreover, it has the potential benefit of being less subjective than radiological evaluation, especially when considering that experts are scarce due to the rarity of these lesions. To enable automated image analysis, recent deep learning-based image segmentation techniques [18–20] have to be applied that precisely delineate lesion voxels, followed by an automated classification stage, e.g., via radiomics [21], which finds discriminative intensity and texture features within a segmented lesion of interest.

**Fig. 1:**
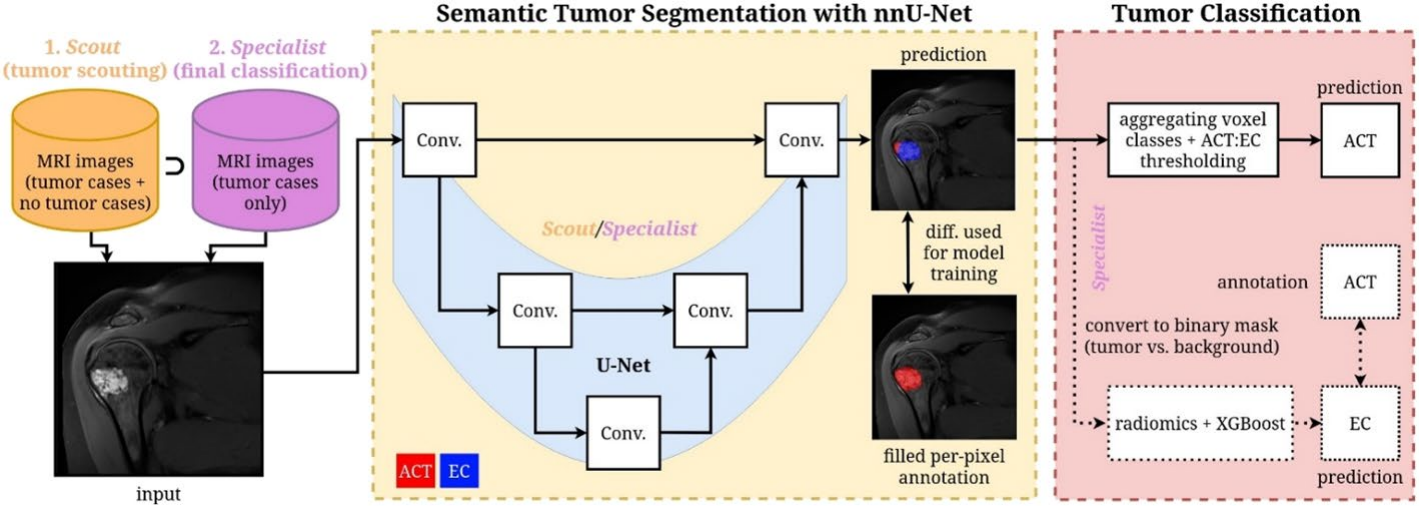
Overview of our proposed AI-based image analysis tool for ACT and EC classification. The pipeline sequentially uses two nnU-Net segmentation models, Scout and Specialist, the former trained on tumor and non-tumor images and the latter trained on tumor cases only. The task of Scout is to detect all no-tumor cases during inference. After eliminating the no-tumor cases, we use Specialist for the final classification. The classification uses voxel class aggregation and ACT:EC ratio thresholding. Alternatively, our tool can perform radiomics-based classification using binarized Specialist predictions

In this study, we therefore aimed at (1) developing and (2) validating an automatic AI-based image analysis tool, trained on expert-provided radiological assessment of cartilaginous tumors, which differentiates EC and ACT on MRI of knee and shoulder based on machine learning, i.e., nnU-Net-based lesion segmentation and radiomics-based classification (see Fig. 1).

## Results

### Segmentation results

Table 1 presents the fivefold cross-validation results for tumor segmentation using our two studied nnU-Net-based models (Scout and Specialist) on EC and ACT datasets.

**Table 1:** Tumor segmentation performance stratified by tumor type for Scout and Specialist models

Model	Metric	Segmentation performance EC (*n* = 104)				Segmentation performance ACT (*n* = 23)			
		Mean	Median	95% CI	Range	Mean	Median	95% CI	Range
Scout	DSC ↑	0.81	**0.91**	[0.75, 0.85]	[0.00, 0.97]	0.83	0.85	[0.75, 0.88]	[0.38, 0.98]
	Prec. ↑	**0.87**	0.95	[0.81, 0.91]	**[0.00, 1.00]**	0.92	**0.97**	[**0.87, 0.96]**	**[0.64, 1.00]**
	Sens. ↑	0.79	**0.91**	[0.73, 0.84]	**[0.00, 1.00]**	0.79	**0.87**	[0.68, 0.86]	[0.24, 0.98]
	MASD ↓ (mm)^a^	1.34	0.18	[0.47, 5.22]	[0.02, 79.86]	1.67	**0.56**	[0.77, 4.94]	[0.04, 18.40]
Specialist	DSC ↑	**0.82**	0.90	**[0.76, 0.86]**	**[0.00, 0.98]**	**0.85**	**0.88**	**[0.79, 0.88]**	**[0.50, 0.95]**
	Prec.↑	**0.87**	**0.96**	**[0.82, 0.91]**	**[0.00, 1.00]**	**0.93**	**0.97**	**[0.87, 0.96]**	[0.59, 1.00]
	Sens. ↑	**0.80**	0.90	**[0.74, 0.84]**	**[0.00, 1.00]**	**0.80**	0.84	**[0.72, 0.86]**	**[0.33, 0.99]**
	MASD ↓ (mm)^a^	**1.22**	**0.11**	**[0.47, 4.35]**	**[0.01, 66.70]**	**1.30**	0.60	**[0.67, 3.44]**	**[0.10, 12.36]**

Holes within tumor annotations were filled using a convex hull-based algorithm. Training used multi-label segmentation, testing used aggregated binary segmentation masks (tumor vs. background). Bold values mark highest performance in the respective metric. 95% confidence intervals (CI) computed using BCa bootstrap (10,000 resamples)

^a^MASD calculated only for cases where both ground truth and prediction contain tumor voxel. EC: 99/104 valid cases for both models (5 empty predictions). ACT: 23/23 valid cases for both models

*Scout*: 17 EC cases and 7 ACT cases had MASD > 1 mm; 2 EC and 1 ACT case had MASD > 5 mm

*Specialist*: 17 EC cases and 6 ACT cases had MASD > 1 mm; 2 EC and 1 ACT case had MASD > 5 mm

Statistical comparison (permutation test, 10,000 iterations): No significant differences found between *Scout* and *Specialist* for EC or ACT tumor segmentation performance (all *p* > 0.05)

*Overall performance*: Both models achieved robust tumor segmentation across tumor classes. Mean Dice Similarity Coefficient (DSC) exceeded 80% for both models on EC and ACT, with Precision consistently above 87% and Sensitivity above or equal to 79%. No statistically significant differences were observed between Scout and Specialist models across any metric (permutation test, two-sided, 10,000 iterations, all p >> 0.05).

*EC vs. ACT Performance*: ACT images yielded slightly higher segmentation performance than EC for both models. Scout achieved a mean DSC of 83% [95% CI 75%, 88%] on ACT versus 81% [75%, 85%] on EC. Similarly, Specialist achieved 85% [79%, 88%] on ACT versus 82% [76%, 86%] on EC. Importantly, both models maintained robust lower confidence interval bounds ≥ 75% DSC across both tumor classes.

*Boundary delineation accuracy*: Mean Absolute Surface Distance (MASD) analysis (122 valid cases where both ground-truth and predictions contained tumor) revealed high precision in tumor boundary delineation. Median MASD values remained below 0.6 mm for both models, indicating excellent agreement with expert annotations. However, mean MASD values (Scout: 1.34–1.67 mm; Specialist: 1.22–1.30 mm) were elevated by three outlier cases with MASD > 5 mm.

### ROC curve analysis

**Fig. 2:**
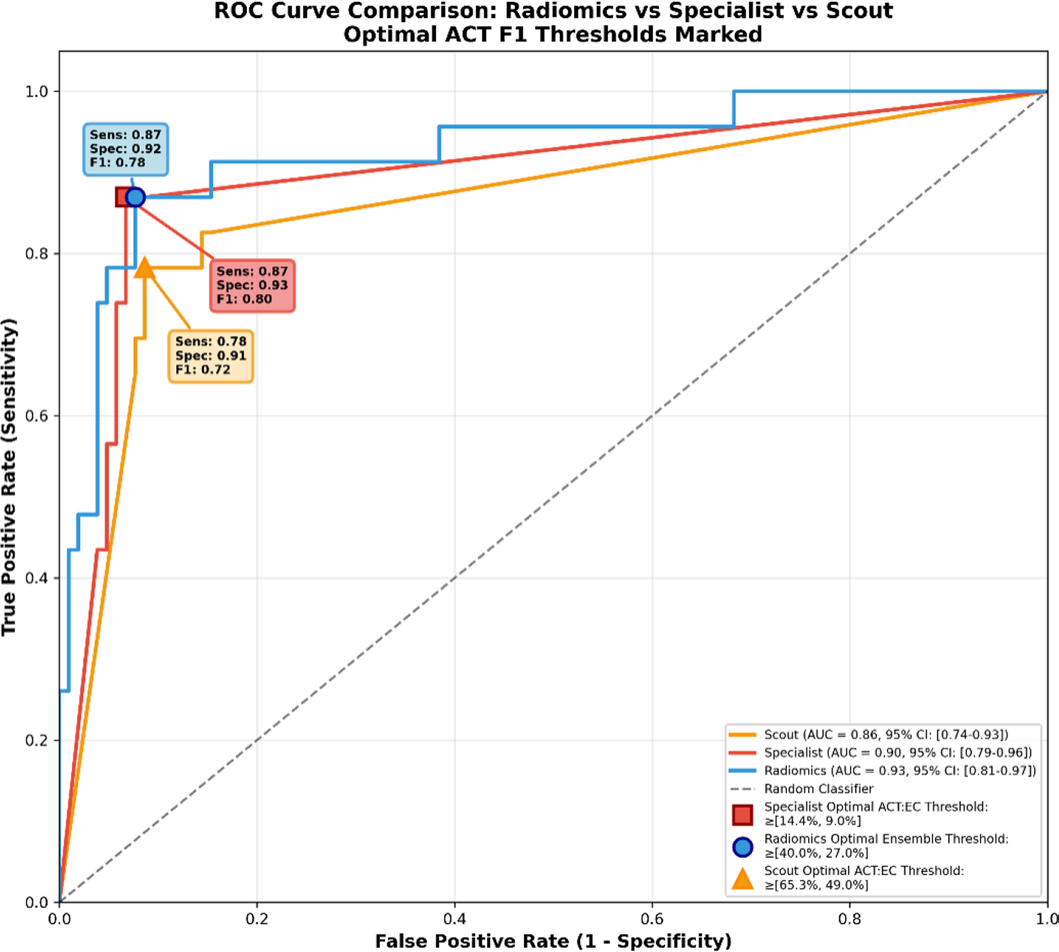
ROC Curve Comparison between Radiomics ensemble and nnU-Net-based Specialist and Scout models. There is no statistical difference between the AUC of the Radiomics ensemble and Specialist model (DeLong, *p* = 0.49). For all classification models, we use the classification threshold that maximizes ACT F1 Score performance, visually marked on each ROC curve. All models outperform the random classifier (0.50 AUC) substantially (Scout: 0.86, Specialist: 0.90, Radiomics: 0.93 AUC)

For all classification models, we performed Receiver Operating Characteristic (ROC) curve analysis to optimize ACT classification performance (ACT F1 score), depicted in Fig. 2. For nnU-Net-based Scout and Specialist models, we optimized the ACT: EC voxel ratio threshold of predicted segmentation masks. For radiomics-based models, we optimized the minimum ACT classification probability threshold.

For the Scout model (Area under the Receiver Operating Characteristic, AUC = 0.86 [0.74, 0.93]), majority voting (≥ 50% ACT: EC voxel ratio) was already within the optimal threshold range identified by ROC analysis [65.3%, 49.0%]. However, for the Specialist model (AUC = 0.90 [0.79, 0.96]), selecting a threshold within the optimal range [14.4%, 9.0%] led to substantial improvement in ACT Sensitivity compared to majority voting (87% vs. 74%). Similarly, for the radiomics ensemble based on ground-truth segmentation masks (AUC = 0.93 [0.81, 0.97]), threshold optimization improved ACT Sensitivity from 57 to 87% by selecting a threshold within the optimal range [40.0%, 27.0%]. There was no statistically significant difference between the AUC of the radiomics ensemble and Specialist model (DeLong test, *p* = 0.49). To reflect real clinical scenarios where ground-truth segmentation masks are unavailable, we also evaluated the radiomics ensemble on Specialist segmentation mask predictions (not shown in Fig. 2 to reduce clutter), which yielded AUC = 0.92 [0.80, 0.96]. This was also not statistically different from the Specialist model (DeLong test, *p* = 0.52). These results on their own should however be interpreted with caution, since due to class imbalance (104 EC vs. 23 ACT cases), high AUC values can be achieved even with suboptimal ACT Precision.

### Tumor scouting classification results

Table 2 presents the tumor discrimination and screening performance of Scout and Specialist models using the optimized ACT:EC voxel ratio threshold that maximized ACT F1 score.

**Table 2:** Performance evaluation of nnU-Net-based tumor classification models in the tumor scouting phase (with ACT, EC, and NT images)

ACT F1 Score optimized models (Based on ROC analysis)		Scout		Specialist	
Class	Metric	Mean	95% CI	Mean	95% CI
EC (*n* = 104)	Precision↑	0.95	[0.89, 0.98]	**0.97**	**[0.92, 0.99]**
	Sensitivity ↑	0.91	[0.85, 0.96]	**0.93**	**[0.87, 0.97]**
	F1 Score ↑	0.93	[0.89, 0.96]	**0.95**	**[0.91, 0.98]**
ACT (*n* = 23)	Precision ↑	0.67	[0.46, 0.83]	**0.74**	**[0.55, 0.89]**
	Sensitivity ↑	0.78	[0.57, 0.92]	**0.87**	**[0.67, 0.96]**
	F1 Score ↑	0.72	[0.55, 0.85]	**0.80**	**[0.64, 0.90]**
NT (*n* = 79)	Precision ↑	**0.93**	**[0.86, 0.97]**	0.93	[0.84, 0.97]
	Sensitivity ↑	**0.86**	**[0.77, 0.93]**	0.78	[0.68, 0.87]
	Specificity ↑	**0.96**	**[0.91, 0.98]**	**0.96**	**[0.91, 0.98]**
	F1 Score ↑	**0.89**	**[0.83, 0.94]**	0.85	[0.78, 0.91]

Both models classify empty predictions (*n*=5) as no-tumor cases. For all models, classification threshold was optimized using ROC curve analysis to maximize ACT F1 Score performance. Bold values mark highest performance in the respective metric. 95% confidence intervals (CI) computed using BCa bootstrap (10,000 resamples)

*EC and ACT classification*: The Specialist model outperformed Scout for both tumor classes, achieving F1 scores of 95% [91%, 98%] vs. 93% [89%, 96%] for EC and 80% [64%, 90%] vs. 72% [55%, 85%] for ACT. ACT classification proved more challenging than EC for both models, with lower precision and sensitivity reflecting the subtle imaging characteristics distinguishing ACT from EC.

*Non-tumor (NT) detection*: Scout demonstrated superior sensitivity for NT detection (86% [77%, 93%] vs. 78% [68%, 87%]), identifying more tumor-free cases while maintaining identical specificity compared with Specialist (96% [91%, 98%]). This higher sensitivity makes Scout more suitable for basic tumor screening, i.e., ruling out non-tumor cases.

Importantly, neither model misclassified any ACT-containing image as non-tumor (NT), demonstrating reliable detection of the semi-malignant tumor variant.

### Final classification results

Table 3 compares the tumor discrimination performance of the Specialist model against the radiomics-based approach (XGBoost classifier ensemble), with both methods trained and evaluated exclusively on tumor-containing images. All models were optimized for high ACT F1 Score performance using ROC curve analysis.

**Table 3:** Performance comparison of tumor classification models on final classification phase (ACT and EC images only): Specialist model (using ratio of ACT to EC voxels for classification), radiomics ensemble evaluated on Specialist segmentation predictions (clinical setting), and radiomics ensemble trained and evaluated on ground truth annotations (with filled holes, ideal scenario)

ACT F1 Score optimized models (Based on ROC analysis)	Class	Precision ↑	Sensitivity ↑	F1 Score ↑
nnU-Net-based specialist model (ACT:EC voxel ratio optimized)	EC	**0.97 [0.92**, **0.99]**	**0.93 [0.87,0.97]**	**0.95 [0.91**, **0.98]**
	ACT	**0.74 [0.55**, **0.89]**	**0.87 [0.67**, **0.96]**	**0.80 [0.64**, **0.90]**
Radiomics features + XGBoost classifier (Ensemble, *Specialist* model predictions labels)	EC	0.95 [0.89, 0.98]	**0.93 [0.87**, **0.97]**	0.94 [0.90, 0.97]
	ACT	0.72 [0.52,0.88]	0.78 [0.57, 0.92]	0.75 [0.59,0.87]
Radiomics features + XGBoost classifier (Ensemble, ground truth segmentation labels)	EC	**0.97 [0.92, 0.99]**	0.92 [0.86, 0.96]	**0.95 [0.91,0.97]**
	ACT	0.71 [0.52,0.87]	**0.87 [0.67**, **0.97]**	0.78 [0.63,0.89]

Empty Specialist segmentation predictions (*n* = 5) were considered as EC predictions. For all models, classification threshold was optimized using ROC curve analysis to maximize ACT F1 Score performance. Bold values mark highest performance in the respective column and class. 95% confidence intervals (CI) computed using BCa bootstrap (10,000 resamples)

*Specialist vs. radiomics (ground truth)*: The Specialist model achieved comparable performance to the radiomics approach for ACT classification, with F1 scores of 80% [64%, 90%] vs. 78% [63%, 89%] and matching ACT Sensitivity of 87% [67%, 96%] vs. 87% [67%, 97%]. The Specialist model demonstrated slightly higher ACT Precision (74% [55%, 89%] vs. 71% [52%, 87%]). For EC classification, both approaches achieved similarly high performance (F1 score: 95%).

*Impact of predicted vs. ground-truth segmentation*: To simulate real clinical scenarios where ground-truth segmentation masks are unavailable, we evaluated the radiomics approach (trained on ground truth) using the Specialist model’s predicted segmentation masks for unseen test images. This realistic scenario resulted in performance degradation across both tumor classes, with F1 scores decreasing from 95 to 94% for EC and from 78 to 75% for ACT. Notably, ACT sensitivity declined from 87% [67%, 97%] to 78% [57%, 92%].

Overall, the nnU-Net-based Specialist model seems beneficial as it solves segmentation and classification end-to-end, eliminating segmentation error propagation inherent to the multi-stage radiomics pipeline. Although the difference in ACT Sensitivity between Specialist (87%) and radiomics tested on Specialist segmentation masks (78%) was not statistically significant (permutation test, two-sided, 10,000 iterations, *p* = 0.38) due to the small sample size, the observed 9-percentage-point difference is highly clinically relevant for detecting ACT tumors.

### Most discriminative radiomics features

**Fig. 3:**
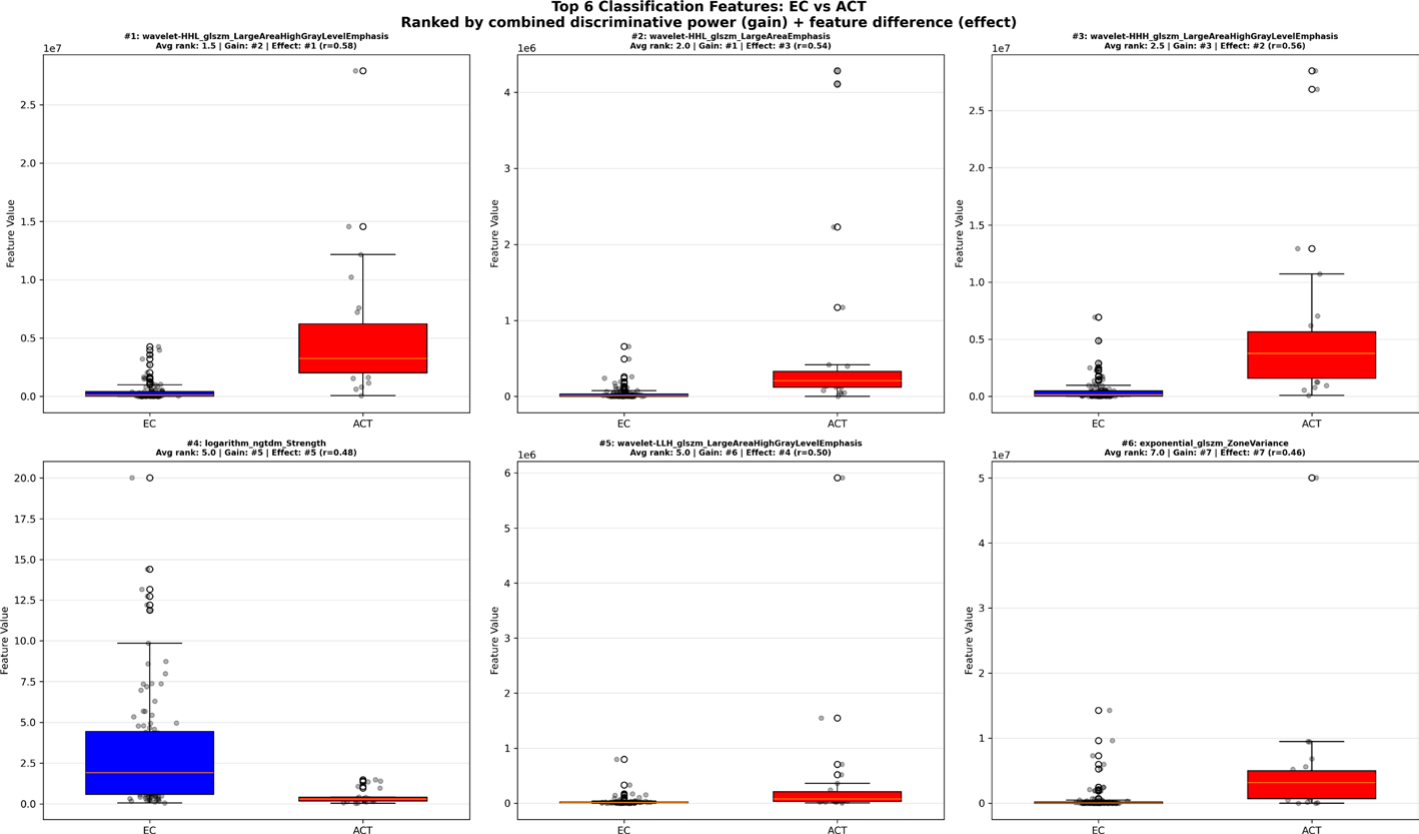
Boxplot of the top 6 Radiomics features grouped by ground-truth tumor class. All features are used within the tumor classification ensemble and are ranked based on their importance in classification (average XGBoost gain) and feature difference (Mann–Whitney *U* test effect size, r). All top features differ significantly by tumor class (*p* < 0.0001)

**Fig. 4:**
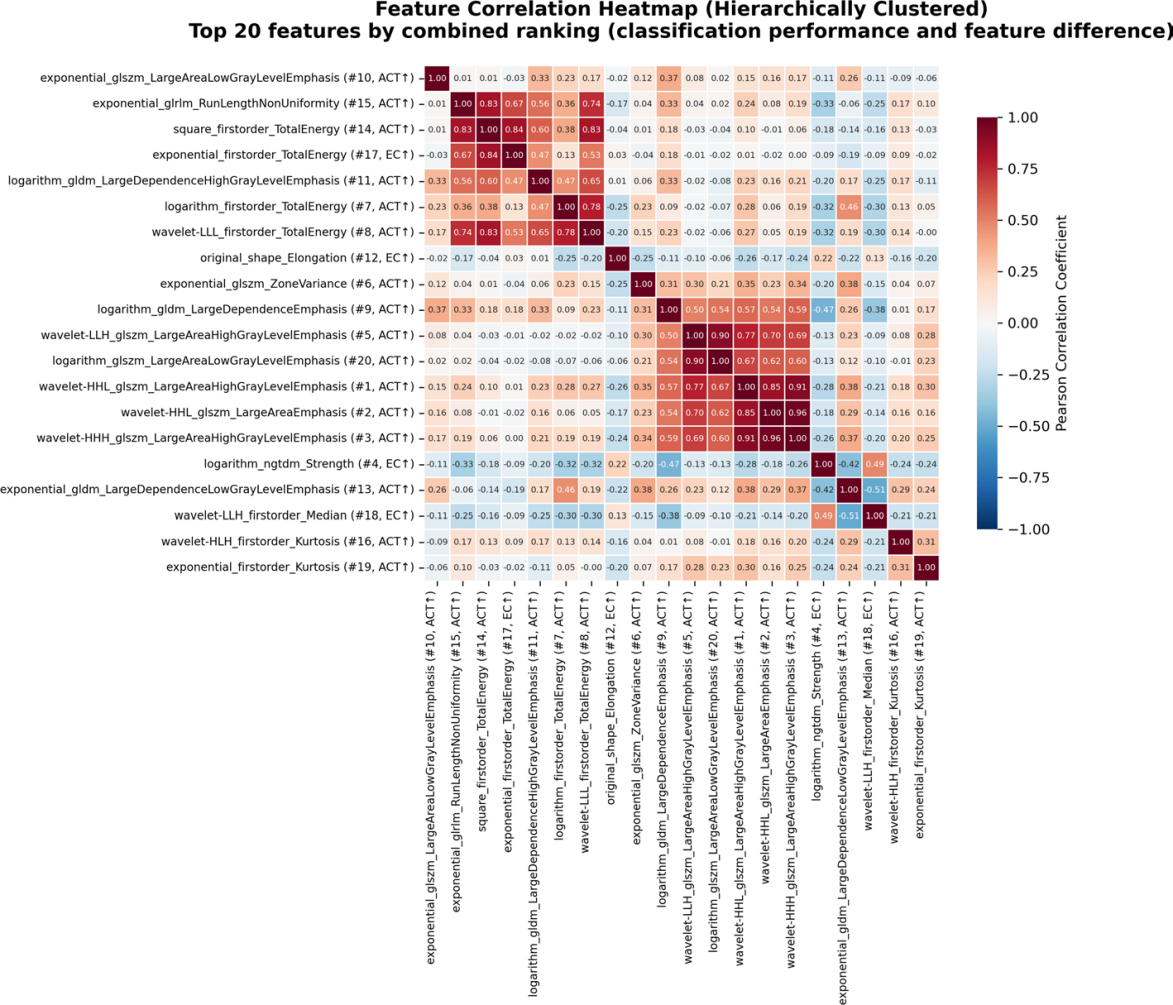
Correlation matrix of the top 20 Radiomics features used in our classification ensemble (out of overall 69 unique features). Features are labeled with their rank and effect direction, determined by classification importance (average XGBoost gain) and effect size (Mann–Whitney U, r). All top features differ significantly by tumor class (*p* < 0.0001). Individual XGBoost models use a Pearson correlation threshold of |0.4|. This matrix identifies robust feature families potentially linked to biological tumor differences

As opposed to the deep learning-based approach, the radiomics approach enables investigation of the explainability of features used for predictions. Our final radiomics ensemble utilized 69 unique features from 1409 extracted radiomics features, selected through variance thresholding, correlation-based redundancy removal (threshold =|0.40| Pearson correlation coefficient), and model-driven selection across 100 independent training iterations. Ranking these features according to their combined importance in classification performance and feature effect size identified a distinctive texture-based signature differentiating enchondroma from ACT, with wavelet-transformed features dominating the top-ranked predictors. All top-ranked features demonstrated significant differences between tumor types (*p* < 0.0001). Figure 3 presents boxplots of the six most discriminative features stratified by tumor type, demonstrating clear separation between EC and ACT classes with minimal overlap (effect sizes *r* = 0.37–0.58), validating the clinical utility of these radiomic biomarkers. Figure 4 displays the correlation matrix of the top 20 features using hierarchical clustering, revealing distinct feature families that cluster based on shared feature information while maintaining complementary discriminative value across the classification ensemble.

Wavelet-transformed features dominated the top-ranked predictors. High-frequency wavelet decompositions (HHL, HHH, HLH) capturing fine-scale heterogeneity were consistently elevated in ACT (ranks #1–3, #5; effect sizes *r* = 0.50–0.58). Gray Level Size Zone Matrix (GLSZM) large area emphasis features were markedly elevated in ACT (effect sizes r = 0.50–0.58). Neighboring Gray Tone Difference Matrix (NGTDM) strength was significantly higher in enchondroma (rank #4; *r* = 0.48). Enchondromas exhibited greater shape elongation than ACT (rank #12; *r* = 0.37). Total energy features (logarithmic transformation) were markedly elevated in ACT (ranks #7–8). Zone variance (rank #6) and kurtosis features (ranks #16, #19) further differentiated ACT from enchondroma.

### Feature robustness assessment

**Fig. 5:**
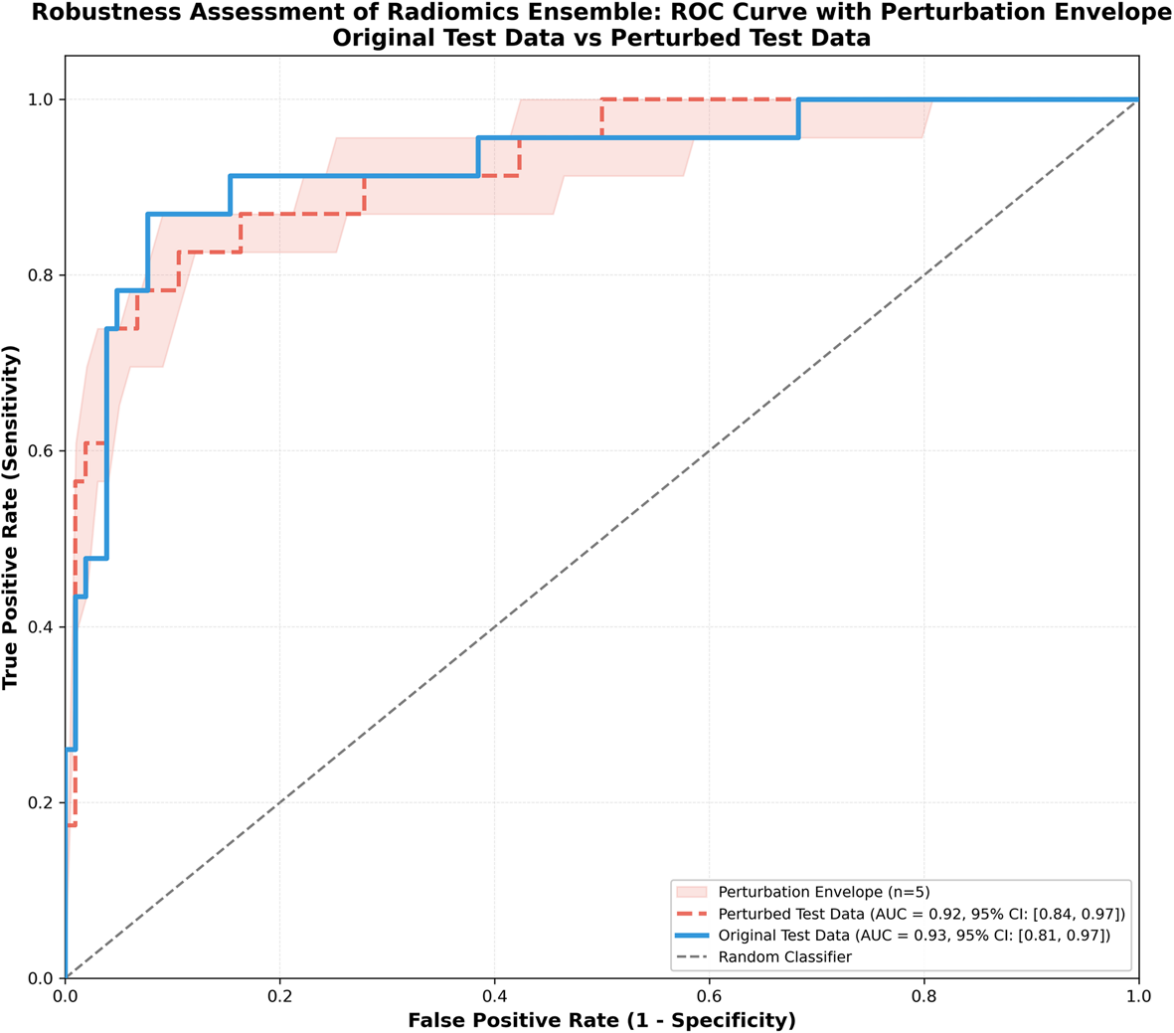
Robustness assessment of Radiomics ensemble showing ROC curves with perturbation envelopes. Five perturbed versions were generated for each test image with Gaussian noise (*σ* = 5% of intensity range) and random rotation ([− 15°, + 15°] around a randomly selected x-, y-, or z-axis). The ground-truth segmentation masks were transformed accordingly. No significant difference was observed between the original test performance and the average perturbed performance (DeLong test, *p* = 0.90)

To also assess the stability of radiomics features to image perturbations, we performed robustness testing on the ensemble model. The corresponding ROC curve with perturbation envelope is shown in Fig. 5. For each test image, five perturbed versions were generated by adding Gaussian noise (*σ* = 5% of intensity range) as well as performing random image rotation (− 15°, + 15°) around a randomly selected x-, y-, or z-axis, with ground-truth segmentation masks transformed accordingly. No significant difference was observed between the original test performance and the average perturbed performance (DeLong test, p = 0.90), demonstrating robust feature extraction and model generalization.

### Qualitative results

**Fig. 6:**
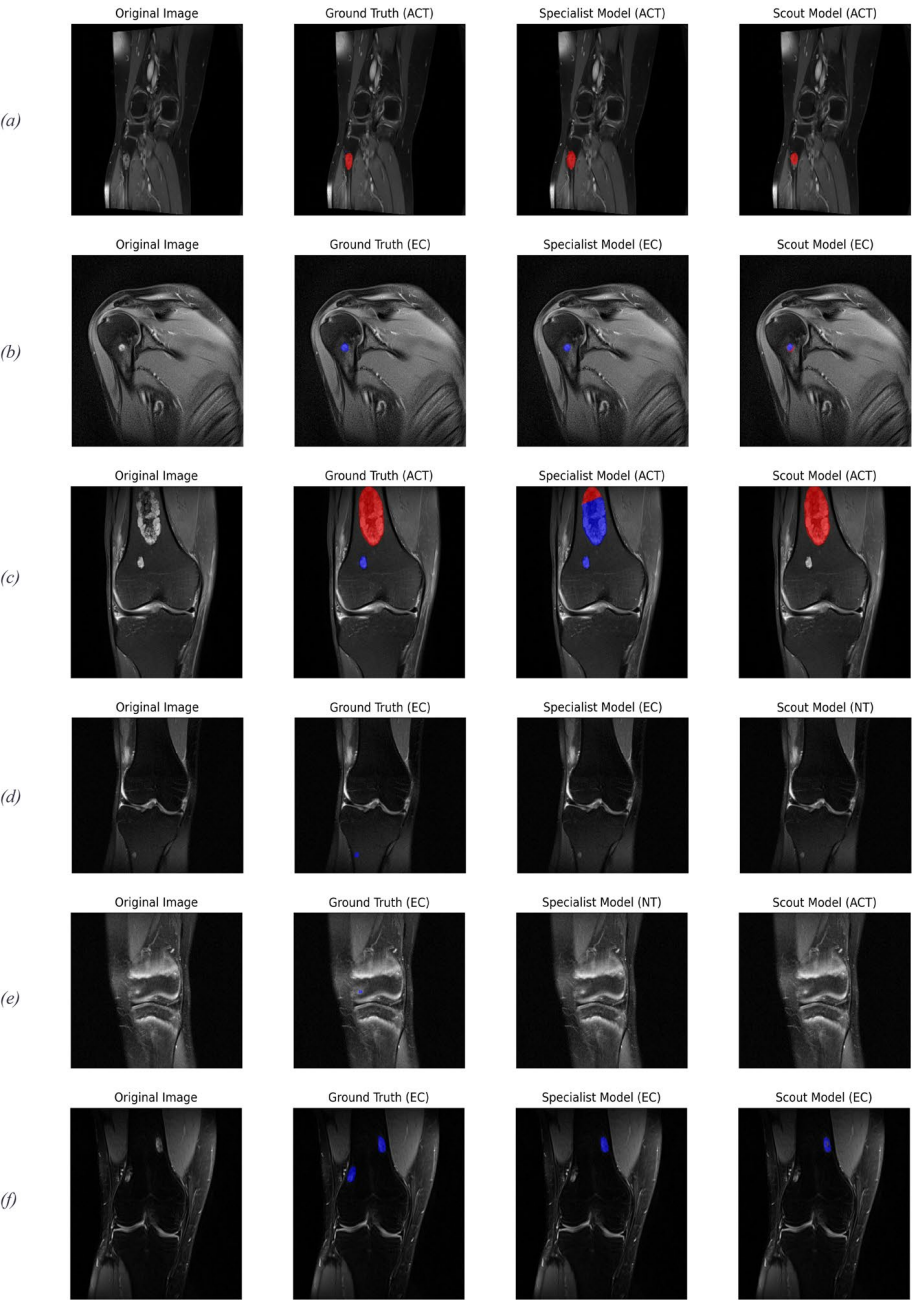
Qualitative comparison between Specialist and Scout models. Each row displays: (left to right) original image, ground-truth segmentation with true class label, Specialist model prediction, and Scout model prediction (small lesions are best seen in digital version). The segmentation color scheme is: blue (EC) and red (ACT). **a**–**c** and **f** demonstrate successful tumor classifications for knee and shoulder images by both models. **c** shows model predictions for a case containing both EC and ACT classes. **d**–**f** illustrate outlier cases with the largest MASD

Figure 6 depicts several example input images and their respective ground-truth and predicted segmentation masks, while also showing the classification outcome of each model in brackets. Figure 6 (a–c) and Fig. 6 (f) show correct classification of knee and shoulder MRI images by both models. Figure 6 (c) shows the results of one of two images that have both tumor classes present and the larger tumor defines the target class. In this case, the Specialist model accurately segmented both tumors, but only labels a part of the ACT correctly. While the Scout model accurately segmented and classified the ACT, it completely omitted the second much smaller EC. The behavior of mixing both tumor classes inside one connected predicted segmentation mask was observed in several images (Fig. 6 b–c). This observation motivated our ROC curve analysis of the ACT:EC voxel ratio threshold parameter.

Figure 6 (d–f) shows the cases that resulted in the highest MASD values for both models. In Fig. 6 (d), the Specialist model incorrectly labeled non-tumor tissue in the middle of the image volume (i.e., generating a false positive on an adjacent slice) achieving 66.70 mm MASD, yet correctly classified the volume as EC, while the Scout model failed to label any voxels as tumor tissue. In Fig. 6 (e), this behavior is reversed, but Scout additionally misclassified the EC as ACT and achieves 79.86 mm MASD. However, both cases feature very small tumor lesions. A third case with outlier segmentation performance, heavily penalized by the MASD metric, is depicted in Fig. 6 (f), where both models successfully segmented only one of two ECs.

## Discussion

In clinical practice, differentiation between EC and ACT is difficult, given that radiological features of both benign and aggressive potential may coexist; experts in this discrimination are scarce due to the rarity of these lesions, and histological analysis is prone to sampling bias [13]. However, accurate differentiation of these lesions is of importance as it dictates further management, from active surveillance with regular MR imaging to surgery.

Our radiomics-based differentiation approach uses hand-crafted features specifically designed for tumor phenotyping, and as expected, it performs well in our small dataset scenario when ground-truth segmentation masks are available while also showing benefits regarding improved explainability of important features. However, in the real-world clinical setting, where tumor segmentation masks have to be predicted instead of being manually annotated, lesion discrimination via direct aggregation of multi-label segmentation predictions outperforms the radiomics-based approach that depends on accurate segmentations by a 9-percentage-point difference in ACT Sensitivity. Despite not being statistically significant, we find this difference clinically relevant. The overall performance of the deep learning-based method is surprising as deep learning-based methods generally require sufficient data to learn robust task-specific feature representations from scratch. We believe that our results can be explained by (1) the end-to-end (segmentation and classification in one model) modeling of our nnU-Net approach, (2) the foreground oversampling, and (3) the strong data augmentation used in nnU-Net, which artificially increases the amount of image variation seen during training. This allows the nnU-Net to converge to a meaningful and robust tumor classifier despite the small imbalanced dataset.

Furthermore, we could show that a sequential approach with two models following a separation of concerns strategy works best. This is implemented with a tumor scouting model to first filter out non-tumor images and a final classification model specialized on tumor images, which leads to a higher tumor classification performance than using a single model.

Hence, the Scout model is better at identifying no tumor cases, whereas the Specialist model has a higher tumor discrimination performance, especially in the more critical ACT class.

### Radiomics feature interpretation

A benefit of radiomics vs. deep learning-based black box approaches is its better explainability of imaging features discriminating EC and ACT. Multi-scale textural heterogeneity emerged as the primary discriminative characteristic. High-frequency wavelet decompositions (HHL, HHH, HLH) capturing fine-scale heterogeneity were consistently elevated in ACT (ranks #1–3, #5; effect sizes *r* = 0.50–0.58), reflecting increased intratumoral complexity at submillimeter spatial scales. These high-frequency wavelet features capture textural irregularities that represent the cumulative imaging manifestations of cellular atypia, infiltrative growth patterns, and irregular tissue boundaries characteristic of ACT's permeative growth within the spongious bone.

Paradoxically, ACT simultaneously demonstrated both fine-scale disorganization and large-scale spatial clustering. GLSZM large area emphasis features, markedly elevated in ACT (effect sizes *r* = 0.50–0.58), indicate the presence of larger homogeneous zones within the tumor architecture. This multi-scale heterogeneity pattern, where ACT exhibits spatial clustering of similar intensities at certain scales (large homogeneous myxoid-rich regions) yet demonstrates fine-scale textural variation at other scales that may reflect cellular infiltration and matrix disruption, revealing a complex hierarchical texture organization that is fundamentally distinct from enchondroma’s rather uniform architecture.

In contrast, enchondromas demonstrated preservation of organized cartilaginous architecture, reflected by significantly higher NGTDM strength (rank #4; *r* = 0.48). This feature measures textural coarseness and primitive pattern strength; higher values in enchondroma indicate mature, organized hyaline cartilage with strong, regular spatial intensity patterns. ACT’s lower strength reflects architectural disruption from cellular infiltration, myxoid degeneration, and loss of the uniform lobular cartilage organization characteristic of benign lesions.

Unexpectedly, enchondromas exhibited greater shape elongation than ACT (rank #12; *r* = 0.37). This finding likely reflects differing growth constraints: enchondromas preferentially extend along the path of least resistance (i.e., within the spongious bone and thus along the bone’s longitudinal axis), producing elongated morphology. Conversely, ACT’s cortical remodeling and deep endosteal scalloping, hallmark features of local aggressiveness, permit more isotropic expansion, resulting in relatively more spherical morphology despite radiologically evident bone erosion.

Intensity-based features further distinguished the two tumor types. Total energy features (logarithmic transformation) were markedly elevated in ACT (ranks #7–8), reflecting increased signal intensity on proton density fat-suppressed imaging. This elevated signal corresponds with increased tissue water content and may reflect myxoid matrix degeneration, where hyaline cartilage matrix undergoes liquefactive changes with accumulation of free water, a hallmark feature more prevalent in ACT, different from the mature, organized cartilage of enchondroma. Zone variance (rank #6) and kurtosis features (ranks #16, #19) further captured the spatial heterogeneity and non-Gaussian intensity distribution characteristic of ACT's mixed composition (regions of preserved cartilage, myxoid degeneration, and increased cellularity).

Notably, these wavelet and second-order texture features substantially outperformed conventional first-order statistics, demonstrating that transformation of cartilaginous tumors into more aggressive variants (i.e., from enchondroma to ACT) manifests primarily as alterations in spatial texture patterns and architectural organization rather than simple intensity changes. The prominence of GLSZM (zone-based), Gray Level Dependence Matrix (GLDM, dependence-based), and NGTDM (neighborhood-based) features suggests that tumor biology in cartilaginous lesions is best characterized by analyzing spatial relationships between voxels rather than their individual intensities. It has to be noted that this interpretation is based on features extracted from manually created ground-truth segmentation masks that are generally not available in clinical practice, though. However, these findings might form a foundation to study the radiological definition of chondrosarcomas in more detail in future work.

### Limitations

First, our ground-truth definition of EC and ACT was based on radiological features and not on histological findings. Given that histological differentiation between EC and ACT is prone to sampling bias, the radiological differentiation using well-defined MRI-based criteria appears valid [13]. Nevertheless, follow-up studies should aim at correlating model predictions with histopathological findings in case patients undergo surgery. Second, owing to the structure of the data used, further clinical details or follow-up images were not available. However, the model allows us to differentiate between EC and ACT on a single image, thus supporting treatment decision-making during the first clinical visit. Third, in some cases, segmentation predictions of EC and ACT were simultaneously present for the same tumor, as seen in Fig. 6, thus complicating the analysis. Yet, this mirrors the evolution of cartilaginous tumors from small lesions with no features of aggressiveness to larger ones with (partial) characteristics of malignancy. To account for this, our model classifies lesions depending on the relative amount of one or the other component based on the ACT to EC voxel threshold, which was optimized with an ROC analysis. This threshold optimization significantly improves the performance of the Specialist model, as seen in Fig. 2. The ROC analysis was estimated over the ensemble results and can be rerun in a computationally efficient manner as new annotated data becomes available to further increase the generalization of the chosen threshold. Fourth, large lesions could always be segmented correctly, while the performance of the model was poorer in smaller-sized lesions. However, all lesions defined as ACT were correctly segmented by the model, implying that even ACTs < 5 cm (e.g., in the fibular head) display features the model correctly captures as such. Finally, we used data from a single radiological center only, where also no potentially confounding findings like plasmacytoma were present. Thus, there is a risk of our model overfitting to our biased training data distribution, especially due to the small size of ACTs (*n* = 23) in the cohort. Therefore, additional external validation studies are required to assess and reproduce the model performance on independent larger and more comprehensive image datasets.

## Conclusions

In this pilot study, we have empirically demonstrated that an AI-based tool can solve the tasks of segmentation and classification of EC and ACT in knee and shoulder MRI scans, despite the small number of annotated training images. The finding that 87% of clinically relevant ACTs were successfully detected in our experiments is promising, so that AI tools may be used for tumor screening of ACT and EC in future clinical practice.

## Materials and methods

### Dataset

**Fig. 7:**
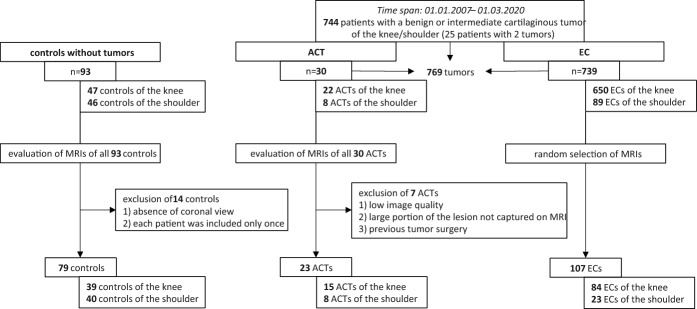
Flowchart indicating selection of patients included in this study

**Fig. 8:**
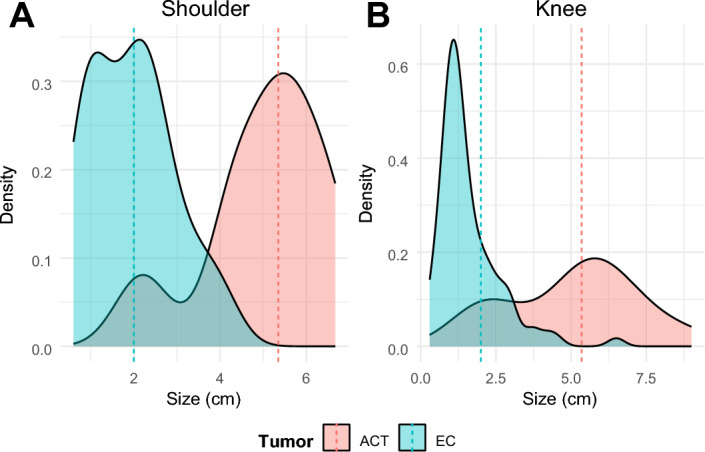
Density plots showing the distribution of tumor sizes in the EC and ACT groups, with dotted vertical lines indicating the median tumor size for each class

The dataset comprises 206 proton-density fat-suppressed MRI scans (79 controls, 103 patients with a single EC, 21 patients with a single ACT, 2 patients with both ACT and EC, and 1 patient with two ECs) of the knee and shoulder regions taken from a retrospective analysis described in [4, 5, 10] that has been approved by the local ethics committee (No. 36–070 ex 23/24). From an initial cohort of 744 tumor patients, all available cases with ACTs (*n* = 23) were included due to their underrepresentation, whereas the remaining tumor cases were randomly selected from patients with ECs. The resulting MRI dataset consists of 104 images containing EC and 23 images with ACTs as described in Fig. 7. Distribution of tumor sizes for both EC and ACT groups is shown in Fig. 8. Notably, two MRIs contain both tumor types. There is also one patient with two ECs, resulting in a total of 107 ECs. The remaining 79 images are from controls and without tumors (NT) and were selected from a pool of 93 control images, with selection limited to coronal view images and one image per patient to avoid over-representation of patients. In all images with multiple lesions, individual tumors were separate and did not form connected components. The mean age of the population at MRI was 51 ± 18 years, and 120 patients (57.4%) were male (Table 4). The composition of the dataset regarding EC and ACT characteristics is shown in Table 5. The MRI volumes have mean dimensions of (318 ± 55, 304 ± 46, 21 ± 4) voxels, with a physical resolution of (0.59 ± 0.11, 0.59 ± 0.11, 4.58 ± 0.68) mm per voxel. More detailed MRI specifications can be found in the supplementary materials. The annotated EC and ACT regions comprise around 0.1% of total voxels, thus leading to class imbalance. Due to the small individual dataset sizes, we combined knee and shoulder images into one dataset, which performed best in preliminary segmentation experiments.

**Table 4:** Characteristics of patients with cartilaginous lesions included in this study

	Total count (*n* = 209)	Control (*n* = 79)	EC (*n* = 107)	ACT (*n* = 23)	p-value^1^
Gender					
Female	89 (42.6%)	27 (34.2%)	49 (45.8%)	13 (56.5%)	0.10*
Male	120 (57.4%)	52 (65.8%)	58 (54.2%)	10 (43.5%)	
Age [years]	51 ± 18	51 ± 19	50 ± 19	57 ± 24	0.07**
Joint					
Knee	139 (66.5%)	40 (50.6%)	84 (78.5%)	15 (65.2%)	< 0.01*
Shoulder	70 (33.5%)	39 (49.4%)	23 (21.5%)	8 (34.8%)	
Side					
Left	108 (51.7%)	44 (55.7%)	51 (47.7%)	13 (56.5%)	0.70*
Right	101 (48.3%)	35 (44.3%)	56 (52.3%)	10 (43.5%)	

^1^comparison of Control, EC and ACT

^*^Chi-squared test

^**^Kruskal–Wallis test

**Table 5:** Characteristics of cartilaginous tumors included

	Total count (*n* = 130)	EC (*n* = 107)	ACT (*n* = 23)	*p*-value^1^
Tumor size [mm]	17 ± 17	14 ± 12	54 ± 20	< 0.01**
Bone				
Humerus (prox.)	31 (23.8%)	23 (21.5%)	8 (34.8%)	0.07***
Femur (dist.)	74 (56.9%)	62 (57.9%)	12 (52.2%)	
Tibia (prox.)	16 (12.3%)	16 (15.0%)	0 (0.0%)	
Fibula (prox.)	8 (6.2%)	5 (4.7%)	3 (13.0%)	
Patella	1 (0.8%)	1 (0.9%)	0 (0.0%)	
Location in relation to medullary canal				
Central	64 (49.2%)	61 (57.0%)	3 (13.0%)	< 0.01***
Peripheral	66 (50.8%)	46 (43.0%)	20 (87.0%)	
Location				
Epiphysis	8 (6.2%)	8 (7.5%)	0 (0.0%)	< 0.01***
Epimetaphysis	14 (10.8%)	11 (10.3%)	3 (13.0%)	
Metaphysis	74 (56.9%)	67 (62.6%)	7 (30.4%)	
Metadiaphysis	14 (10.8%)	8 (7.5%)	6 (26.1%)	
Diaphysis	19 (14.6%)	12 (11.2%)	7 (30.4%)	
Patella	1 (0.8%)	1 (0.9%)	0 (0.0%)	
Periosteal reaction				
Yes	0 (0.0%)	0 (0.0%)	0 (0.0%)	
No	130 (100.0%)	107 (100.0%)	23 (100.0%)	
Medullary edema				
Yes	2 (1.5%)	0 (0.0%)	2 (8.7%)	0.03***
No	128 (98.5%)	107 (100.0%)	21 (91.3%)	
Endosteal scalloping				
No	116 (89.2%)	106 (99.1%)	10 (43.5%)	< 0.01***
Superficial	4 (3.1%)	1 (0.9%)	3 (13.0%)	
Deep	10 (7.7%)	0 (0.0%)	10 (43.5%)	
Reason for referral				
Tumor-associated	21 (16.2%)	8 (7.5%)	13 (56.5%)	< 0.01*
Other reason	74 (56.9%)	69 (64.5%)	5 (21.7%)	
Unknown	35 (26.9%)	30 (28.0%)	5 (21.7%)	
Contrast agent				
Yes	19 (14.6%)	7 (6.5%)	12 (52.2%)	< 0.01*
No	111 (85.4%)	100 (93.5%)	11 (47.8%)	
Number of received MRIs	1 ± 1	1 ± 1	1 ± 1	0.08**

^1^comparison of EC and ACT

^*^Chi-squared test

^**^Wilcoxon rank-sum test

^***^Fisher’s exact test

### Reference annotation of segmentation and classification labels

The free annotation software ITK-SNAP (version 3.8.0) [22] was used for manual tumor segmentation. ITK-SNAP outputs NIFTI files that contain mask information delineating the cartilage lesions for each MRI sequence. Manual tumor annotations were performed by one of the coauthors (J.W.), who had conducted an extensive literature review and received specialized training in the imaging characteristics of cartilaginous tumors. All annotated lesions were reviewed and verified by two experienced orthopedic oncologists (M.S. and A.L.). Diagnosis of EC or ACT was made based on the following radiological criteria: tumor size larger than 4.9 cm, deep endosteal scalloping (i.e., involvement of more than 2/3 of bone cortex), tumor-related adjacent medullary edema, periosteal reaction, as previously described [10]. In case one of the criteria that are highly suggestive of an aggressive lesion was positive, the lesion was diagnosed as ACT [10–13, 23]. Image evaluation was first carried out by an experienced coauthor with special training for image interpretation of EC vs. ACT (J.W.), followed by reexamination by an expert orthopedic oncologist (A.L.).

### Image analysis study design

As depicted in Fig. 1, our AI-based image analysis tool addresses two distinct tasks: semantic voxel-wise segmentation of tumors in each patient volume and patient-wise classification of segmented tumors for EC and ACT differentiation. The 3D segmentation task performs separation of tumor tissue from surrounding anatomical structures in MRI images. Given our limited dataset size (MRI images: 104 EC, 23 ACT, and 79 without tumor) and the high imbalance between semi-malignant and benign tumor occurrence as well as the small size of tumors in the images, we investigated machine learning methods specifically designed for medical imaging tasks with small annotated training sets and class imbalance. Therefore, we used the state-of-the-art nnU-Net framework [24, 25] (V2) for semantic image segmentation. nnU-Net has become the standard framework for semantic segmentation in medical imaging due to its ease of use, while still maintaining state-of-the-art segmentation performance [26] on various datasets (including tumor datasets like BRATS [27] in brain imaging). The patient-wise classification task distinguishes between three classes: No Tumor (NT), ACT, and EC. Regarding image classification, we evaluate two strategies: (1) directly aggregating the per voxel votes of the nnU-Net predicted segmentation masks (i.e., multi-label segmentation), thus performing simultaneous segmentation and classification, and (2) an ensemble of XGBoost classifiers [28] trained on radiomics features [29] derived within binarized lesion segmentation masks. These radiomics features are specifically designed to potentially capture the phenotypic texture characteristics of tumors, thus promising accurate and explainable results in the presence of small training data sets.

### Semantic segmentation and classification with nnU-Net

Traditionally, for multi-label segmentation of distinct EC and ACT, we would need to manually design a machine learning solution, including model selection, deep neural network architecture, training duration, and hyperparameters. This process is both time-intensive and error-prone. The deep learning-based nnU-Net framework [24] addresses this challenge by condensing expert knowledge in medical image segmentation in the form of heuristics derived from an automated dataset preanalysis phase, thus delivering a standardized pipeline.

As shown in Fig. 1, our methodology employs two distinct nnU-Net models: a Scout model trained on the complete dataset (encompassing knee and shoulder images with tumor, as well as non-tumor cases), and a Specialist model trained exclusively on annotated knee and shoulder tumor images. This approach follows a separation of concerns strategy, where the Scout model performs an initial screening to identify and filter out tumor-free images (tumor scouting). Subsequently, the Specialist model is applied to the remaining cases for definitive classification, allowing it to focus specifically on distinguishing between different tumor types (final tumor classification). All nnU-Net models were trained with V2 using the default settings, the 3D high-resolution architecture, and without residual encoder for 1,000 epochs per fold.

While nnU-Net primarily focuses on multi-label voxel-level segmentation, we also extended it to perform image-level (i.e., patient-wise) classification by aggregating the voxel-wise segmentation mask predictions. Specifically, we calculate the ratio of predicted ACT to EC voxels to determine the classification label of the entire image. Images without any tumor-labeled voxels are classified as no-tumor cases. We optimize the threshold of the ratio of ACT:EC voxels used for classification through Receiver Operating Characteristic (ROC) curve analysis.

### Classification with radiomics

nnU-Net relies on deep learning for extracting task-specific image segmentation features directly from data. In contrast, radiomics [29] features are mathematically well-defined image processing features that are used to describe the radiographic phenotype of a tumor, extracted from a region of interest (ROI) or volume of interest (VOI) defined by a segmentation mask—in our case, annotated or predicted tumor regions.

After feature extraction using PyRadiomics [29], we train an ensemble of XGBoost classifiers [28] to distinguish between EC and ACT classes using these extracted radiomics features. XGBoost implements gradient tree boosting, which excels at handling structured tabular data, like our radiomics features, as shown in [28, 30]. The supplementary materials contain a detailed description of our radiomics tumor classification approach, including feature extraction specifications and a completed METRICS [31] questionnaire that quantifies the quality of our radiomics approach.

### Radiomics feature importance quantification

To assess explainability of radiomics features for predictions, we employed a dual-ranking approach to comprehensively assess feature importance of the final radiomics ensemble, combining both discriminative power and feature difference between tumor types. First, XGBoost gain importance was calculated across all trained models, quantifying each feature's contribution to classification accuracy through its cumulative reduction in model loss during tree-based splitting. Second, we computed effect sizes (r) using Mann–Whitney U tests to assess the magnitude of difference between EC and ACT groups. Features were independently ranked by both metrics, and a combined ranking was generated through weighted averaging (50% XGBoost rank + 50% effect size rank), with lower combined ranks indicating superior features. Only features achieving statistical significance (Mann–Whitney *U* test, two-sided, *p* < 0.05) were retained for final ranking. This allows us to identify features that are both highly discriminative for machine learning classification and biologically meaningful in their magnitude of difference between tumor types, thus establishing improved explainability of predictions compared with a deep learning-based classification approach.

### Statistical analysis

Our experimental validation methodology builds upon two foundations: the established validation protocol from the nnU-Net framework [24], which has been extensively tested across various medical image datasets, and the recent recommendations from the Metrics Reloaded framework [32]. The latter addresses common shortcomings in evaluation metric selection for biomedical image analysis, which can impede successful clinical translation.

nnU-Net evaluation: Models trained for multi-class segmentation underwent fivefold cross-validation following nnU-Net’s protocol, where each fold used 80% of data for training and 20% for validation, ensuring each image was tested exactly once. We combined all out-of-fold predictions to compute final performance metrics.

Radiomics evaluation: For the radiomics approach using binary segmentation masks (tumor vs. background) derived from multi-class nnU-Net predictions, we exploited the computational efficiency of XGBoost to run 100 randomized trials. In each trial, we used stratified 80/20 train-test splits to maintain class balance. We combined all 100 trial models into a classification ensemble, where each model only classifies images from its held-out test set, yielding a prediction for every image comparable to the nnU-Net cross-validation setup. While a similarly comprehensive evaluation would be desirable for nnU-Net, it is computationally prohibitive due to significantly longer training times (× 42 in our setting). For both approaches and all metrics, 95% confidence intervals (CI) were computed using scipy’s bootstrap implementation based on the bias-corrected and accelerated (BCa) bootstrap algorithm to quantify test sample uncertainty.

### Evaluation metrics

Based on the recommendations of [32], we used two different groups of metrics for segmentation and classification. For segmentation, we combined the widely used Dice Similarity Coefficient (DSC) as a counting metric with the Mean Absolute Surface Distance (MASD) as a distance-based metric. For classification, we combined one-vs-all counting-based metrics (F1 score, Sensitivity, Specificity, Precision) with a multi-threshold metric (AUC) evaluated on the semi-malignant ACT class, as we are most interested in having a high True Positive Rate (TPR). Furthermore, we use AUC for evaluation despite the common view that Area Under the Precision–Recall curve is preferable for imbalanced datasets. Recent research [33] demonstrates that AUC remains more meaningful in classification settings where false negatives carry higher costs, such as in cancer screening.

## Supplementary Information

Supplementary Material 1.

## Data Availability

All derived data and materials used in this publication are available on request.

## Abbreviations

ACT: Atypical cartilaginous tumor
AI: Artificial intelligence
AUC: Area under the receiver operating Characteristic
EC: Enchondroma
CS: Chondrosarcoma
DSC: Dice similarity coefficient
GLDM: Gray level dependence matrix
GLSZM: Gray level size zone matrix
MASD: Mean absolute surface distance
MRI: Magnetic resonance imaging
NGTDM: Neighboring gray tone difference matrix
NT: No tumor
ROC: Receiver operating characteristic
TPR: True positive rate
