# Supplementary material for: Machine learning vs. radiomics for discriminating atypical cartilaginous tumors from benign enchondromas on MRI
Source: Biomed Eng Online. 2026 Mar 24;25:66. doi: 10.1186/s12938-026-01547-0 (PMC13134191; doi:10.1186/s12938-026-01547-0)
Supplement: Supplementary file 1 — Supplementary Material 1. [file 12938_2026_1547_MOESM1_ESM.docx]

**SUPPLEMENTARY MATERIALS**

**Machine learning vs radiomics for discriminating atypical cartilaginous tumors from benign enchondromas on MRI.**

**Authors:**

Simon Johannes Joham^1,*^, Johannes Nikolaus Woltsche2,*, Dieter Szolar3, Andreas Leithner2, Martin Urschler1,#, Maria Anna Smolle2

**Affiliations:**

1 Institute for Medical Informatics, Statistics and Documentation, Medical University of Graz, Graz, Austria

2 Department of Orthopaedics and Trauma, Medical University of Graz, Graz, Austria

3 Diagnostikum Graz, Graz, Austria

*these authors contributed equally and thus share first authorship

**^#^Corresponding Author:**

Assoc. Prof. Priv.-Doz. Dr. techn. Dipl.-Ing. Martin Urschler

Institute for Medical Informatics, Statistics and Documentation, Medical University of Graz, Graz, Austria

address: Auenbruggerplatz 2/V, 8036 Graz, Austria

email: [martin.urschler@medunigraz.at](mailto:martin.urschler@medunigraz.at)

phone: +43 316 385 13587

**Radiomics feature extraction and model development**

Extraction software and ROI definition: Radiomics features were extracted using PyRadiomics version 3.0.1 with default configuration settings. Features were computed from 3D volumetric MRI images in per-patient fashion, with regions of interest (ROIs) defined by ground-truth segmentation masks. To ensure anatomically contiguous tumor volumes, all holes within segmentation masks were filled using a convex hull algorithm prior to feature extraction.

*Image pre-processing*

PyRadiomics 3.0.1 default settings were applied throughout the extraction pipeline. This included:

- No image resampling (native MRI spacing preserved; resampledPixelSpacing = None)
- No interpolation applied
- No global intensity normalization (normalize = False; native MRI intensity values used)
- Fixed bin width discretization (default method) with bin edges equally spaced from zero
- Dimensionality: All features were extracted in 3D mode (force2D = False) from volumetric data

*Feature categories*

The extraction pipeline with all image types and all features enabled (enableAllImageTypes() and enableAllFeatures()) generated 1,409 radiomic features across eight filter/transformation types:

- Original features (n=107): First-order statistics, shape, and texture features from unfiltered images
- Wavelet-transformed features (n=744): Features from 8 wavelet decompositions (LLL, LLH, LHL, LHH, HLL, HLH, HHL, HHH)
- Filtered features (n=558 total): Exponential (n=93), Gradient (n=93), Local Binary Pattern 2D/3D (n=93), Logarithm (n=93), Square (n=93), and Square Root (n=93)

Note: LBP-2D and LBP-3D refer to different image filter implementations (not extraction dimensionality), both of which were included as they are separate image types enabled by default in PyRadiomics.

*Feature selection pipeline*

To ensure reproducibility and prevent overfitting, we implemented a two-stage feature selection strategy applied independently within each of 100 train-test splits:

Stage 1 - Low-Variance Removal: Features with variance less than 0.01 in the training set were removed using VarianceThreshold to eliminate uninformative features.

Stage 2 - Redundancy Removal (Multicollinearity Control): Highly correlated features were iteratively removed using Pearson correlation with threshold of 0.40. For each correlated pair (absolute correlation greater than 0.40), the feature with lower variance was removed. This threshold was chosen to be more stringent than typical recommendations (0.85-0.95) to maximize feature independence. The algorithm iteratively identified correlated pairs in descending order of correlation magnitude, removing one feature from each pair until no correlations exceeded the threshold. Final verification confirmed maximum pairwise correlation below |0.40| in all retained feature sets.

Feature stability across splits: Individual models utilized 20-30 features per trial depending on the specific train-test split. Across all 100 trials, 69 unique features appeared in at least one model, demonstrating reasonable feature stability while allowing model diversity through different train-test compositions.

*Feature normalization and model training*

Normalization: After feature selection, StandardScaler (z-score normalization) was applied independently to each train-test split, with parameters fit on training data only and applied to test data to prevent data leakage.

Model architecture: XGBoost binary classifier with hyperparameters optimized via 3-fold stratified cross-validation grid search on training data. The search space included: n_estimators (100, 200, 300), max_depth (2, 3, 4), learning_rate (0.01, 0.1), subsample (0.5, 1.0), colsample_bytree (0.5, 1.0), L1 regularization reg_alpha (0.5, 1.0), and L2 regularization reg_lambda (0.5, 1.0).

Model validation strategy: 100 independent train-test splits with 80/20 ratio and stratified sampling to maintain original class distribution. All feature selection and hyperparameter optimization were performed exclusively on training data using 3-fold cross-validation. Performance was evaluated on held-out test sets. The final ensemble model aggregated predictions across all 100 independently trained models.

*METRICS scoring adherence*

Our pipeline adheres to key METRICS criteria:

- Feature extraction software specified (PyRadiomics 3.0.1 with default settings, IBSI compliant)
- ROI definition clear (manually segmented 3D tumor volumes with convex hull filling)
- Pre-processing documented (default PyRadiomics settings: no resampling, no normalization, fixed bin width discretization)
- Feature selection method explicit (variance filtering with threshold 0.01, correlation-based redundancy removal with Pearson correlation threshold 0.40)
- Multicollinearity addressed (pairwise Pearson correlation below |0.40| enforced through iterative removal)
- 3D computation specified (volumetric per-patient analysis, force2D = False)
- Model validation rigorous (100 independent train-test splits with proper train-test isolation, 3-fold cross-validation for hyperparameter tuning)
- Performance metrics comprehensive (AUC, Sensitivity, Specificity, Precision, and F1 score for both classes with bootstrap confidence intervals)
- Feature robustness assessed (perturbation analysis with noise injection and random rotation)

**MRI specifications**

All machine-learning models were trained exclusively on coronal proton-density fat-suppressed (PD-FS) sequences, while clinicians had access to all of the following MRI sequences during image evaluation.

Knee MRI examinations were performed on two 3T scanners (Magnetom Skyra and Magnetom Vida; Siemens Healthcare Diagnostics GmbH, Austria) using either a 15- or 18-channel dedicated knee coil. The imaging protocol consisted of coronal, transverse, and sagittal PD-FS sequences, as well as a coronal T1-weighted turbo spin-echo (TSE) sequence. Coronal PD-FS images were acquired with a field of view (FOV) of 160/140 mm, a matrix of 307×384, TR 3000/3200 ms, TE 34/25 ms, and a slice thickness of 3 mm with a 0.6-mm interslice gap. Transverse PD-FS sequences used an FOV of 160/150 mm, a 307×384 matrix, TR 5460/4110 ms, TE 37/35 ms, and a slice thickness of 2.5–3/3 mm with a 0.6-mm gap. Sagittal PD-FS images were acquired with an FOV of 160/140 mm, a 307×384 matrix, TR 2920 ms, TE 34 ms, and a slice thickness of 3 mm with a 0.6-mm gap. Coronal T1-weighted TSE images were obtained using an FOV of 160/140 mm, matrices of 346×384 or 290×484, TR 690 ms, TE 11/19 ms, a slice thickness of 3 mm, a 0.6-mm gap, and a flip angle of 180°/150°.

Shoulder MRI examinations were also conducted on the same two 3T systems using a 16-channel shoulder coil. The protocol comprised five sequences: coronal PD-FS, sagittal proton-density or T2-weighted TSE without fat suppression, transverse PD Dixon TSE with fat suppression, and both non-contrast and post-contrast coronal T1-weighted TSE sequences. Coronal PD-FS images were acquired with an FOV of 160/150 mm, matrices of 320×320 or 256×256, TR 2250 ms, TE 46/49 ms, and a slice thickness of 3 mm with a 0.3/0.9-mm gap. Sagittal PD/T2 TSE images used an FOV of 160/130 mm, matrices of 384×384 or 272×320, TR 3520/2490 ms, TE 28/37 ms, and a slice thickness of 3 mm with a 0.9/0.6-mm gap. Transversal PD Dixon TSE sequences were acquired with an FOV of 160/140 mm, a 256×256 matrix, TR 2250/3120 ms, TE 43/50 ms, and a slice thickness of 3/3.5 mm with a 0.3/1-mm gap. Coronal T1-weighted TSE images without fat suppression used an FOV of 150/140 mm, matrices of 403×448 or 269×384, TR 574/598 ms, TE 12/11 ms, and a slice thickness of 3 mm with a 0.3/0.9-mm gap. The post-contrast coronal T1-weighted TSE sequence with fat suppression was acquired with similar FOV and matrix settings (150/140 mm; 256×320 or 269×384), TR 660/550 ms, TE 11 ms, and a slice thickness of 3 mm with a 0.9-mm gap.
